# Pro-senescence neddylation inhibitor combined with a senescence activated β-galactosidase prodrug to selectively target cancer cells

**DOI:** 10.1038/s41392-022-01128-2

**Published:** 2022-09-09

**Authors:** Shuaishuai Ni, Qian Liu, Xin Chen, Lele Ding, Lili Cai, Fei Mao, Donglei Shi, Robert M. Hoffman, Jian Li, Lijun Jia

**Affiliations:** 1 Cancer Institute, Longhua Hospital, Shanghai University of Traditional Chinese Medicine, 200032 Shanghai, China; 2 State Key Laboratory of Bioreactor Engineering, Shanghai Frontiers Science Center of Optogenetic Techniques for Cell Metabolism, Frontiers Science Center for Materiobiology and Dynamic Chemistry, Shanghai Key Laboratory of New Drug Design, School of Pharmacy, East China University of Science and Technology, 200037 Shanghai, China; 3 Department of Surgery, University of California, San Diego, CA USA

**Keywords:** Chemical biology, Drug delivery

**Dear Editor**,

Targeting senescence therapy is a promising anticancer strategy by arresting the cell-cycle and inducing cellular senescence. However, senescent cells can promote malignancy and drug resistance.^1,2^ Thus, with the induction of senescence emerged as a viable therapeutic concept, it is important to consider how to eliminate those senescent cancer cells.

Senescent cells are vulnerable to particular small-molecule inhibitors, known as “senolytics” which trigger apoptosis preferentially in senescent cells. Recently, a two-step senescence-focused therapeutic concept was proposed that combines a senescence inducer with a senolytic drug to selectively eliminate senescent cells.^3^ Theoretically, the senescence-focused combination strategy not only increases the anticancer effect and reduces the risk of resistance, but also decreases the superposed safety risk of classical drug combinations. However, low pro-senescence selectivity of inducers and scarcity of senolytic compounds trammel the application of two-step senescence-focused anticancer strategy. Hence, apart from continuous discovery of specific inducers and senolytics, it is necessary for us to improve the strategy of targeting senescence combination therapy.

Protein neddylation is an important posttranslational modification that conjugates NEDD8 (neural precursor cell expressed, developmentally down-regulated gene 8) to substrates, such as cullin family (skeleton proteins of cullin-RING E3 ligases). We and other studies previously identified that a small-molecule neddylation inhibitor, MLN4924 (MLN), induced cancer cellular senescence by suppressing the p21 degradation.^4,5^ Herein, several independent experiments were conducted to further evaluate the pro-senescence activity and selectivity of MLN.

Senescence associated β-galactosidase (SA-β-gal) is a classical senescence marker. An SA-β-gal staining assay showed that MLN had effectively pro-senescence activity in multiple cancer cell lines, comparable with other representative senescence inducers (Fig. 1a; Supplementary Fig. 1a, b). Furthermore, MLN induced more SA-β-gal in cancer cells compared to normal cells, especially in A549 and H1299 human lung cancer cell lines than in BEAS-2B human lung cells (Fig. 1b; Supplementary Fig. 1b). Next, two additional experiments, including an ONPG color assay and an SA-β-gal responsive fluorescence imaging, visually showed the pro-senescence selectivity of MLN (Supplementary Fig. 2a, b). BCL2 inhibitor navitoclax, known as a specific senolytic drug, is used to identify novel senescence inducers. The present study showed that navitoclax eliminated the MLN-induced senescent A549 cells with less affecting the non-senescent cells (Supplementary Fig. 2c). Western blotting showed that both A549 and H1299 cells were accumulated more p21 than BEAS-2B cells, by the treatment of MLN (Supplementary Fig. 3a, b). Meanwhile, MLN induced more SA-β-gal in A549 cells than in p21-silenced A549 cells (Supplementary Fig. 3c, d), suggesting that the pro-senescence selectivity of MLN was related to the accumulation of p21.

**Fig. 1:**
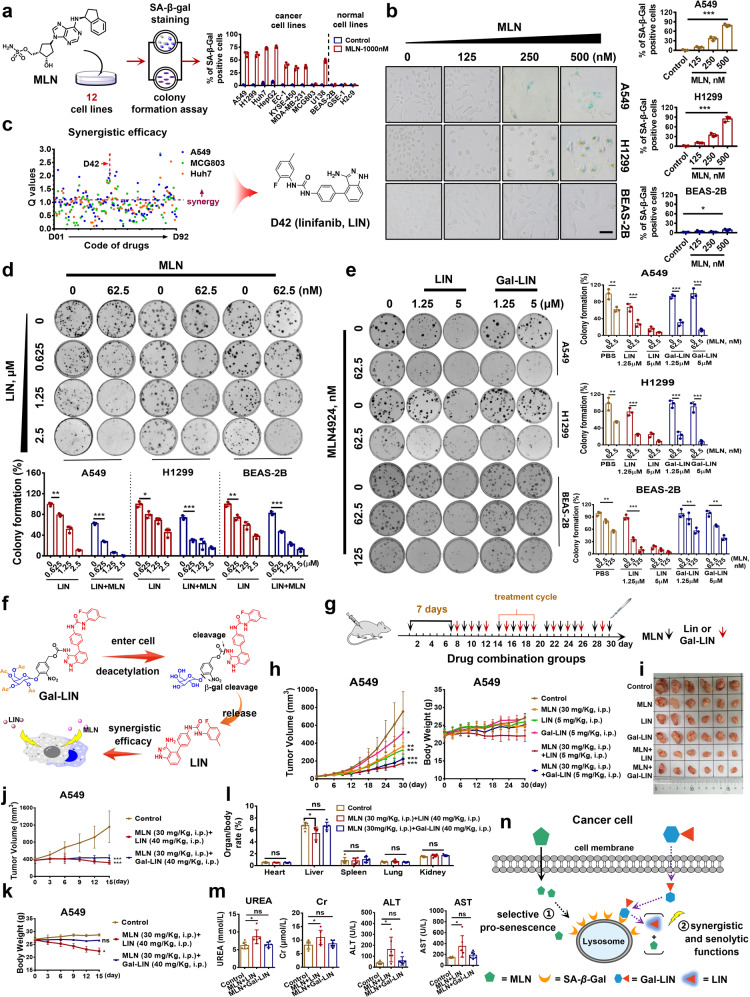
MLN-driven bifunctional prodrug combination strategy is an improved senescence-focused anticancer combination therapy. **a** Schematic depicting the pro-senescence evaluation process of MLN. **b** Evaluation of the pro-senescence activity of MLN in lung cancer cell lines A549, H1299 and normal lung cell line BEAS-2B by SA-β-gal staining. The results are representative of three independent experiments. Bar graphs represent ratio of SA-β-gal positive cells. Scale bar: 20 μm. **c** Synergistic efficacy of 92 approved anticancer drugs with MLN in A549 (blue), Huh7 (orange), and MCG803 (green) cell lines. Q represents the value of combination efficacy, values >1.15 indicate synergy, values <0.85 antagonism, values at 0.85–1.15 indicate an additive effect; Structure of optimal synergistic drug linifanib (LIN). **d** Colony formation assay and statistical data to compare the synergistic antiproliferative effect of LIN in combination with MLN on A549, H1299 and BEAS-2B cell lines. **e** Colony formation assay and statistical data to compare the antiproliferative activities of LIN or Gal-LIN in combination with MLN on A549, H1299 and BEAS-2B cell lines. **f** Proposed activation mode of Gal-LIN to release LIN by MLN-induced SA-β-gal. **g** Schematic representation of combination administration on A549 bearing mice. Six different regimens, including mock group, three single-administration and two combination-administration groups, were performed with a metronomic schedule. The mice in two combination groups were treated with MLN in advance for 7 days, and then alternately combined with either LIN or Gal-LIN, for six days with an one-day rest as the treatment cycle. **h** Tumor volume change during treatment. The combination of MLN and Gal-LIN exhibited better tumor growth inhibition percentage (TGI) than either drug alone (70.49% vs 30–50%), comparable with the combination of MLN and LIN (76.53%) (left). Mice body weight change during treatment (right). **i** Images of tumors from mice at the 30th day after initiation of treatment. **j** Tumor volume change during treatment. Three regimens, including mock group, the combination group of MLN with LIN or Gal-LIN, were performed with a metronomic schedule. Once tumors reached an average volume of ~400 mm^3^, mice were treated with MLN in advance for 3 days, and then treated with either LIN or Gal-LIN for 12 days. **k** Mice body weight change during treatment. The combination of MLN and LIN caused a significant decrease of the weight ratio of liver to body (*P* < 0.05). **l** Organ/body weight rate after 15 day of treatment. **m** Liver and kidney functional tests; UREA; Cr creatinine, ALT alanine aminotransferase, AST aspartate. **n** Schematic illustration of MLN-induced β-gal activated prodrug delivery as a two-step senescence-focused combination therapy. Statistical significance was calculated with unpaired two-tailed Student’s *t* test. *P < 0.05, **P < 0.01, ***P < 0.001, n.s. indicates no significant difference. Data are mean values ± SEM (*n* = 3)

Subsequently, we explored the practicability of MLN-driven senescence-focused combination therapy, by combining with an SA-β-gal responsively synergistic prodrug, aiming to increase the anticancer effect and decrease the combination toxicity. Ninety-two FDA-approved anticancer drugs were evaluated the synergistic effect with MLN in multiple cancer cell lines (Fig. 1c; Supplementary Fig. 4a, b). Multi-targeting inhibitor linifanib (LIN) presented superior synergistic efficacy (all values of Q = 1.7–2.81, Supplementary Table 1). Combination index (CI) analysis validated the synergy of LIN with MLN (ED_50_ of CI = 0.58–0.67, Supplementary Fig. 5a). Moreover, a colony formation assay showed that the combination of MLN and LIN had more significant growth suppression than either drug alone (Fig. 1d).

Next, an acetylated β-galactosidic prodrug of LIN, Gal-LIN, was designed and synthesized to evaluate the anticancer efficacy in combination with MLN. Gal-LIN remained inactivation but became active when the galacto-conjugation was cleaved off by SA-β-gal (Supplementary Fig. 5b). A colony formation assay showed that the combination of MLN and Gal-LIN not only displayed more effective than either drug alone, but also selectively suppressed the growth of A549 and H1299 cells, including at high concentration of each drug (Fig. 1e). A checkboard test showed that the combination of MLN and Gal-LIN had higher synergy in cancer cells, compared to normal cells (Supplementary Fig. 5c, d). Senolytic drugs have two classical characteristics, including the elimination of senescent cells and apoptotic induction. Gal-LIN significantly decreased the SA-β-gal levels in MLN-induced senescent cells, and induced apoptosis (Supplementary Fig. 5e–g), suggesting that Gal-LIN, as a bifunctional prodrug, not only had a synergistic efficacy with MLN, but also displayed the potential to eliminate senescent cancer cells.

We then investigated the stability and activation mode of Gal-LIN. No significant decomposition of Gal-LIN was observed in PBS with a long incubating time (Supplementary Fig. 6a, b). An additional plasma stability assay showed that Gal-LIN had a moderate stability (T_1/2_ = 50.8 min) in mouse plasma (Supplementary Fig. 6c). Unexpectedly, Gal-LIN was activated slowly with the incubation of single β-gal in vitro (Supplementary Fig. 6a). We hypothesized that the acetyl group of galacto-conjugation possibly weakened the affinity of Gal-LIN with β-gal, thereby decelerating the LIN releasing. To verify the hypothesis, the other enzyme, ester hydrolases, that has a specific deacetylative effect, was incubated with the mixture of β-gal and Gal-LIN. Whole time evaluation assay indicated that dual enzymatic mix quickly activated Gal-LIN to release LIN, compared to these single ones (Supplementary Fig. 6b). Therefore, potential activation mode was proposed that Gal-LIN was firstly deacetylated in cancer cells, and then activated by MLN-induced SA-β-gal to release LIN (Fig. 1f). Intriguingly, LIN had a short-time fluorescence response, but Gal-LIN failed to inherit this characteristic (Supplementary Fig. 6d). Utilizing the difference, we could visually evaluate the activation mode by comparing the fluorescence imaging in cells. The results showed that the fluorescence signals of Gal-LIN treated groups were detected in senescent A549 and H1299 cells induced by MLN.

Finally, the combination of MLN and Gal-LIN was explored the potential on the A549 xenograft tumors in nude mice (Fig. 1g; Supplementary Fig. 7a–c), and exhibited significant tumor growth inhibition (Fig. 1h, i). Previous studies showed that the oncogenic p-AKT was overactivated by MLN, while LIN could effectively inhibit the AKT phosphorylation. The present study showed that the p-AKT overactivated by MLN was suppressed by the combination treatment of either LIN or Gal-LIN (Supplementary Fig. 7d, e). Histological analysis showed that the combination of MLN and Gal-LIN significantly increased TUNEL positivity and decreased SA-β-gal positivity, compared to the combination of MLN and LIN (*P* < 0.05 in TUNEL and *P* < 0.01 in SA-β-gal, Supplementary Fig. 7f–j), suggesting that Gal-LIN selectively eliminated senescent cancer cells induced by MLN in vivo.

We further investigated the efficacy of the combination of MLN and Gal-LIN against advanced tumor, along with the safety profiles. Obviously, the prodrug combination forcefully suppressed the growth of advanced tumor with low toxicity (Fig. 1j–m). To visually evaluate the MLN-driven prodrug activation process in vivo, we synthesized a theranostic molecular Gal-DOX. In comparison with other tested groups, the Gal-DOX-treated group specifically enhanced the relative fluorescence intensity in the MLN pre-treatment tumor (Supplementary Fig. 7k), validating that these galacto-conjugated prodrugs were selectively activated in tumor with the combination of MLN.

In summary, the strategy of MLN-driven senescence-focused prodrug combination therapy displayed a promising anticancer effect and low toxicity (Fig. 1n). We proposed that the strategy might be expanded to other types of combinations, such as MLN combining with β-gal activated cytotoxic agents or senolytic drugs. Besides, the development of MLN-driven prodrug combination therapy has considerable promise for improving cancer treatment, as well as other human senescence-related disorders.

## Supplementary information

Pro-senescence neddylation inhibitor combined with a senescence activated β- galactosidase prodrug to selectively target cancer cells

## Data Availability

The authors declare that the data supporting the findings of this study are available within the paper and its Supplementary materials.
