# Supplementary material for: Pro-senescence neddylation inhibitor combined with a senescence activated β-galactosidase prodrug to selectively target cancer cells
Source: Signal Transduct Target Ther. 2022 Sep 9;7:313. doi: 10.1038/s41392-022-01128-2 (PMC9458665; doi:10.1038/s41392-022-01128-2)
Supplement: Supplementary file 1 — Pro-senescence neddylation inhibitor combined with a senescence activated β- galactosidase prodrug to selectively target cancer cells [file 41392_2022_1128_MOESM1_ESM.doc]

Supplementary Materials for

Pro-senescence neddylation inhibitor combined with a senescence activated β-galactosidase prodrug to selectively target cancer cells

Shuaishuai Ni^1, †^, Qian Liu^1, †^, Xin Chen^2, †^, Lele Ding^1, †^, Lili Cai^1^, Fei Mao^2^, Donglei Shi^2^, Robert M. Hoffman^3^, Jian Li^2^, *, and Lijun Jia^1^, *

**Author affiliations and contact information**

^1^Cancer Institute, Longhua Hospital, Shanghai University of Traditional Chinese Medicine, Shanghai, 200032, China.

^2^State Key Laboratory of Bioreactor Engineering, Shanghai Frontiers Science Center of Optogenetic Techniques for Cell Metabolism, Frontiers Science Center for Materiobiology and Dynamic Chemistry, Shanghai Key Laboratory of New Drug Design, School of Pharmacy, East China University of Science and Technology, Shanghai, 200037, China.

^3^Department of Surgery, University of California, San Diego, California, USA

^†^These authors contributed equally to this work.

***Corresponding author**: Lijun Jia, Email: ljjia@shutcm.edu.cn; Jian Li, Email: jianli@ecust.edu.cn

**This PDF file includes:**

Materials and Methods

Figures. S1 to S8

Table. S1

Supplementary Material of Figure S1

**MATERIALS AND METHODS**

**Materials**

Human lung cancer cell lines A549 and H1299; human liver cancer cell lines Huh7 and HepG2; human esophageal carcinoma cell lines EC-1 and KYSE-450; human breast cancer cells MDA-MB-231; human gastric cancer cells MCG803; human glioma cancer cells U138, and three normal cell lines, including human normal lung cells BEAS-2B; human normal gastric cells GSE-1; rat cardiacmyoblast H2c9 were obtained from the American Type Culture Collection (Manassas, VA) and Insitute of Biochemistry and Cell Biology (CAS Shanghai). Cells were cultured in Dulbecco’s modified Eagle’s medium (Hyclone, Logan, UT), containing 10% fetal bovine serum (Biochrom AG, Berlin, Germany) and 100 units/ml penicillin/streptomycin solution and maintained in a humidified atmosphere of 5% CO_2_ at 37°C (standard culture conditions). Doxorubicin, palbociclib, etoposide, XL413 and alisertib were purchased and were dissolved in dimethyl sulfoxide (DMSO) and kept at -20°C for *in vitro* studies. MLN4924 and other compounds (such as LIN, Gal-LIN, DOX, Gal-DOX) were dissolved in 5% 2-hydroxypropyl-β-cyclodextrin *in vivo* study. The solution of these drugs were freshly made when was used.

**Synthesis and characterization of Gal-LIN**

The solution of 4-(hydroxymethyl)-2-nitrophenol (1.83 g, 10 mmol, 1 eq.) in acetonitrile (CH_3_CN) (20 mL) stirred at room temperature with 2, 3, 4, 6-tetra-O-acetyl-α-D-galactopyranosyl-bromide (4.93 g, 12 mmol, 1.2 eq.). After stirring for 5 min, silver oxide (6.93 g, 30 mmol, 3 eq.) was added afterwards into the reaction system for 4 hours. The reaction solution was filtrated and washed with CH_3_CN (5 mL). The solvent was removed by the rotary evaporation without further purification. The residue was resolved in dichlormethane (DCM) (20 mL). The solution fell to 0 ^o^C, then added 4-nitrophenyl chloroformate (2 g, 10 mmol, 1 eq.) and pyridine (1 mL) afterwards stirred under a nitrogen atmosphere for 2 hours. The solvent was removed by rotary evaporation and purified by silica gel column chromatography (eluent, EtOAc/petroleum ether, 1:5, V/V) to yield intermediate **1** as a colorless oil. ^1^H NMR (400 MHz, DMSO-*d*_6_) δ 8.28 (d, 2H), 7.92 (s, 1H), 7.66-7.63 (dd, 1H), 7.43 (d, 1H), 7.40 (d, 2H), 5.57 (dd, 1H), 5.50 (d, 3H), 5.31 (d, 2H), 5.17-5.15 (m, 2H), 2.20-2.03 (m, 12H). To a solution of **1** (664 mg, 1.0 mmol, 1eq.) in anhydrous DCM (10 mL) was added LIN (375 mg, 1.0 mmol, 1eq.) and DMAP (19 mg, 0.2 mmol, 0.2eq.) at 0 ^o^C under a nitrogen atmosphere. The reaction mixture was stirred at room temperature for 4 hours. The solution was poured into ice water containing saturated sodium bicarbonate (Na_2_CO_3_). The mixture was then extracted with DCM. The combined extracts were washed with brine, dried over anhydrous Na_2_SO_4_, filtered, and condensed at 30^o^C. The crude was purified by silica gel column chromatography (eluent, EtOAc/petroleum ether, 1: 3, V/V) to yield Gal-LIN as a light yellow solid. Overall yield: 44%; Purity 98.2%; ^1^H NMR (400 MHz, DMSO-*d*_6_) *δ* 9.28 (s, 1H), 8.57 (s, 1H), 8.08 (s, 2H), 8.02 (d, 1H), 7.87 (d, 1H), 7.64-7.58 (m, 3H), 7.48 (d, 1H), 7.42 (d, 2H), 7.18-7.09 (m, 2H), 6.82 (m, 1H), 5.62 (d, 1H), 5.45 (s, 2H), 5.38 (s, 1H), 5.27-5.20 (m, 4H), 4.50 (t, 1H), 4.14 (m, 2H), 2.28 (s, 3H), 2.16 (s, 3H), 2.05 (s, 3H), 2.03 (s, 3H), 1.96 (s, 3H). ^13^C NMR (150 MHz, DMSO-*d*_6_) *δ* 170.43, 170.34, 170.02, 169.36, 152.67, 152.60, 151.54, 150.54, 149.57, 148.78, 140.64, 140.17, 136.80, 134.88, 134.03, 131.65, 131.46, 129.99, 127.38, 125.36, 125.10, 121.39, 118.51, 118.25, 116.25, 115.12, 113.39, 99.01, 71.28, 70.37, 68.12, 67.54, 66.72, 61.69, 21.25, 20.97, 20.86, 20.80, 20.78. HRMS (ESI) m/z calcd C_43_H_41_FN_6_O_15_ [M+Na]^+^ 923.2512, found 923.2511.

**Scheme 1.** Synthetic route of Gal-LIN

|  |
| --- |

*Reagents and conditions*: (a) Ag_2_O, acetonitrile, rt, 4h, 80%; (b) 4-Nitrophenyl chloroformate, pyridine, methylene dichloride, 0 ^o^C to rt, 2h, 90%; (c) 4-dimethylaminopyridine, methylene dichloride, rt, 4h, 60%.

**Cell proliferation and cell clonogenic assays**

For evaluating the proliferation of cultured cells, cells seeded in 96-well plates with 2000 cells per well, in triplicate, and cultured overnight were treated with MLN4924 or other drugs for 72 hours, followed by cell counting kit-8 (CCK-8) assay. The absorbance of each well at 450 nm was recorded using a Microplate Reader (Bio-Tek Instruments, Synergy H1). The cell viability was evaluated by measuring the absorbance at 450 nm and calculated by the formula (cell viability = (OD_positive_ - OD_control_) / (OD_negative_ - OD_control_)). For clonogenic assays, cells were seeded in six or twelve-well plates (150 or 50 cells per well, respectively) in triplicate, and were treated with MLN4924 or other drugs and cultured for 10~14 days. Colonies of control groups were considered as 30~50 cells or more and counted. The representative results of three independent experiments with similar trend were presented.

**SA-*β*-galactosidase staining**

SA-*β*-Gal staining was performed in either cells or tissue cryosections (tumor xenografts, hearts, livers, lungs, kidneys), using the SA-*β*-Gal Staining Kit (Beyotime, C0605). Cells were treated with MLN or other senescence inducers for 72 hours. The expression of SA-*β*-Gal was determined with the SA-*β*-Gal Staining kit according to the manufacturer’s specifications. Briefly, tissue cryosections were fixed at room temperature for 45 min, respectively, with a solution containing 2% formaldehyde and 0.2% glutaraldehyde in PBS. Then, these samples were washed three times with PBS, and incubated overnight at 37°C with the SA-*β*-Gal Staining Kit.

**ONPG chromogenic reaction**

Cells A549, H1299, or BEAS-2B was exposed to the indicated concentrations of MLN4924 (1, 10, 100, 1000nM) or 0.1% (v/v) DMSO for 24h, 48h or 72h, respectively. Cells were washed three times with ice-cold PBS, and then repeated freezing and thawing from -80°C to room temperature for releasing SA-*β*-Gal. After incubating with *β*-gal Assay Kit (Thermo Scientific) according to the manufacturer’s specifications, the samples were centrifuged at 1,500 *g* for 5 min, and taken the upper supernatant tested by UV spectrophotometry (Thermo NanoDrop 2000). The SA-*β*-Gal activity was evaluated by measuring the absorbance of the supernatant of those samples at 405 nm.

**NIR-emitting fluorescent probe KSL11**

The fluorescence imaging studies of cells were observed with a Leica TCS SP8 SMD confocal microscope. Cells (A549, H1299, BEAS-2B) were plated on a glass-bottomed cell culture dish (NEST Biotechnology, 801001) and incubated with normal medium for 1-2 days. Then, the medium was replaced with serum-free medium containing 10μM probe KSL11. The cells were incubated over 20-60 min for different dyes at 37°C and then washed 3 times with PBS before fluorescence imaging experiments with confocal laser scanning microscopy (Leica).

**Western blot analysis**

Cells cultured on 10cm dishes were harvested, rinsed twice with PBS, and then were lysed in RIPA lysis buffer (strong) (YEASEN, 20101ES60), and incubated on ice for 30min. Cell debris was removed by centrifugation at 15,000rpm for 10min at 4°C. The protein concentration of the supernatant was determined with Thermo Fisher protein assay dye reagent (Thermo Fisher). Equal amounts of each sample were mixed with 2 × SDS-PAGE protein loading buffer (YEASEN, 20315ES05), denatured by heating at 95 °C for 10 minutes, then were separated by 8~15% PAGE Gel Quick Preparation Kit (YEASEN, 20324ES62) and transferred to PVDF membrane. The membrane was blocked using 5% nonfat dry milk for 1h at room temperature, followed by overnight incubation at 4 °C with antibodies against target proteins: NEDD8 (Cell Signaling Tec., 2745), cullin2 (abcam, ab166917), p21 (abcam, ab109520), clever-PARP (C-PARP) (Cell Signaling Tec., 5625), clever-caspase3 (C-caspase3) (Cell Signaling Tec., 9661), β-actin (Cell Signaling Tec., 3700). Next, the membrane was incubated with species-specific HRP-conjugated secondary antibody (Anti-rabbit IgG antibody, CST, 7074; Goat anti-Mouse IgG antibody, Arigo, ARG65350). The protein bands were visualized using a chemiluminescence imaging system (Tanon4600SF). ImageJ 1.50 software was used to quantify the band intensity. Each experiment was performed in triplicate.

**Gene silencing using small interfering RNA of p21**

The siRNA oligonucleotides were transfected into cells by using Lipofectamine 2000. Briefly, Opti-MEM (Invitrogen) was used to incubate with siRNA and Lipofectamine 2000 separately for 5 min at room temperature and mixed for 20 min, and then the mixture together with the serum-free medium were applied to the cells (final concentration of siRNA is 20 nM). All siRNAs were synthesized by GenePharma (Shanghai, China). The sequences of the sip21 were as follows: negative control 5′-UUC​UCC​GAA​CGU​GUC​ACG​UTT-3′; sip21 5′-GAC CAU GUG GAC CUG UCAC-3′.

**Drug screening**

As shown in **Fig. S4a**, a robust screen assay was firstly established in a 96-well plate. A549, Huh7 or MCG803 cells were plated at a density of 2000 cells per well, respectively. 24 h later, ninety-two tested anticancer drugs were added to wells, individually. The dose of single tested drug was maintained at the range of 20~40% of inhibitory ratio as the final concentration. The final concentration was preliminary at 1μM, and then adjusted by the double dilution according to the inhibitory ratio. MLN followed to combine with MLN at a 125nM final concentration (inhibitory ratio at 30~40%) for 72 h, and then testified by the cell counting kit-8 (CCK-8) assay. The synergistic efficacy (Q value) was further calculated by Jin's formula. If all Q values of a tested drug were bigger than 1.15 in three cancer cells, this tested drugs was identified as a potential synergistic drug. Jin's formula was used to analyse the effect of drug combination.

$$\text{Q}=\text{E}_{\text{A}+\text{B}}\text{/（E}_{\text{A}}+\text{E}_{\text{B}}\text{-E}_{\text{A}}\times\text{E}_{\text{B}}\text{）}$$

Q represents the value of combination efficacy. Herein, E_A+B_ represents cellular inhibitory ratio of combination; E_A_ represents cellular inhibitory ratio of drug A; E_B_ represents cellular inhibitory ratio of drug B; Values > 1.15 indicate synergy, values < 0.85 antagonism, values 0.85~1.15 indicate an additive effect.

**Combination index and isobologram analysis**

Calculation of combination index (CI) and isobolograms with the CalcuSyn software was described previously. Briefly, CI values were calculated according to the levels of growth inhibition (fraction affected) by each agent individually and combination of MLN with LIN. Isobolograms, which indicate the equipotent combinations of different dose (ED_50_, ED_75_ and ED_90_, etc.), were used to illustrate synergism (CI < 0.9), antagonism (CI > 1.2) and additivity (0.9 < CI < 1.2). Constant ratio combinations of the two drugs (LIN (0.315-5μM) and MLN4924 (31.5-500nM)) at 0.1 of their ED_50_ was used.

**HPLC analysis of release ability of Gal-LIN**

Gal-LIN (10μM) was incubated in PBS (pH = 7.4, 37 ^o^C), PBS (pH = 7.4) with (1 U/mL) β-gal (Sigma, G5635), PBS (pH = 7.4) with (2 U/mL) esterase, PBS (pH = 7.4) with (1 U/mL) β-gal and (2 U/mL) esterase and monitored by HPLC at t = 0, 2, 4, 6, 8, 10 and 12 h, or 0, 20, 40, 60, 80, 100 and 120min. The reaction mixture was analyzed by HPLC at the indicated times. The reaction mixture (200 μL) was mixed with the ice-cold methanol (500 μL), and the resulting solution was placed at -80 ^o^C for 1 hour to precipitate proteins in the reaction mixture. After centrifugation, the supernatant was collected and passed through a 0.22 m filter, and 20 μL of each sample was loaded onto Agilent ZORBAX SB-C18, reversed-phase column (5 μm, 4.6×150mm) on an Agilent 1100 series HPLC system. The column was eluted with methanol/CH_3_CN/water (80:10:10, v/v). The flow rate was set at 0.5 mL/min^-1^. An UV/vis detector was used to monitor the products at 254 nm. Retention times: LIN (7.88 min), Gal-LIN (10.52 min).

**Mice plasma stability**

0.1 M potassium phosphate buffer was preheated with 5 mM MgCl_2_ (K/Mg-buffer), pH 7.4±0.1. Plasma preparation: Thaw frozen plasma by placing at 37 ℃ quickly. Test compounds and reference compounds spiking solution: 0.5 mM compound spiking solution A: 5 µL of 10 mM compound stock solution was added to 95 µL ACN. 0.01 mM compound spiking solution B: 20 µL of spiking solution A was added to 980 µL of 0.1 M K/Mg-buffer. Pre-warm the plasma and spking solution B (see step 3.2) at 37 ℃ for 5 min. 90 µL of pre-warmed plasma was added into the wells designated for all the time points (0, 15, 30, 60, 120, 240, 480 min). For 0 min, 10 µL of spiking solution B was added, and then 400 µL of ACN containing internal standard (IS) was added to the wells of 0-min plate. For other time points, 10 µL of pre-warmed spiking solution B was added into the wells designated for the time points 0, 15, 30, 60, 120, 240, 480 min). At 5, 15, 30, 60,120, 240 min, 400 µL of ACN containing IS was added to the wells of corresponding plates, respectively to stop the reaction. After quenching, the plates was shaked for 5 min (600 rpm) and stored at -20 ℃ (if necessary) until analyzed by LC/MS/MS. 100 μL of the supernatant was transfered from each well into a 96-well sample plate containing 100 μL of ultra pure water for LC/MS analysis, and the area ratio of either Gal-LIN or LIN was counted.

**Optical properties of LIN and Gal-LIN**

UV-Vis spectra were determined on a U-2910 spectrometer (Hitachi). Fluorescence spectra were acquired on an F-4700 fluorescence spectrometer (Hitachi) with a 10-mm quartz cuvette. Either LIN or Gal-LIN was dissolved in dimethyl sulfoxide (DMSO) to prepare stock solutions and then diluted in PBS buffer to a final concentration for measurements. The limit of detection of twelve probes was determined from the fluorescence titration data based on an acknowledged method.

**Checkboard test and quantitative analysis of drug synergy**

Drug synergism was analyzed by CompuSyn software (version 1.0) (http://www.combosyn.com), which is based on the median-effect principle (Chou) and the combination index-isobologram theorem (Chou–Talalay). CompuSyn software generates combination index (CI) values, where CI < 0.75 indicates synergism, CI = 0.75-1.25 indicates additive effects, and CI > 1.25 indicates antagonism. Following the instruction of the software, drug combinations at non-constant ratios were used to calculate the combination index in our study. For calculating the fold change in sensitivity to MLN, the concentration of either LIN or Gal-LIN that inhibited cell proliferation by 50% (GI_50_) was determined for a panel of cancer cell lines A549 and H1299 or normal cell line BEAS-2B in the absence or presence of MLN. Experiments were performed independently twice.

***In vivo* anticancer effects by the metronomic therapy**

Male nude mice were subcutaneously injected with 1.5×10^6^ A549 cells in 100 μL PBS according to protocols of tumor transplant research. After tumor induction (about 14 days, tumor volume is at 40-80 mm^3^), nude mice were divided into six groups (six mice per group) randomly, including mock group with saline (100 μL qd), MLN-treated (30 mg/Kg qd) and LIN-treated (5 mg/Kg qd) groups as two positive controls, Gal-LIN group (5 mg/Kg qd), the same dose of MLN combined with LIN or Gal-LIN. Compounds were administrated by intraperitoneal injection. All groups were treated with one dose per day. Body weights and tumor volumes were measured every 3 days. In the first 7 days, two combination groups were treated with MLN alone for the senescence induction based on the evaluation of MLN pro-senescent time, and then alternately treated with LIN or Gal-LIN in combination (per six days to rest one day). On the 30th day after inoculation, all the mice were sacrificed. Tumor volumes were measured for each group.

***In vivo* efficacy for advanced tumor and subacute toxicity testing**

Once tumor volume is over 350 mm^3^, nude mice were divided into three groups randomly (six mice per group) , including mock group with saline (100 μL qd), combination group of MLN (30 mg/Kg qd) and LIN (40 mg/Kg qd), combination group of MLN and Gal-LIN (40 mg/Kg qd). Compounds were administrated by intraperitoneal injection. All groups were treated with one dose per day. Body weights and tumor volumes were measured every 3 days. In the first 3 days, two combination groups were treated with MLN alone, and then alternately treated with Lin or Gal-LIN in combination (per six days to rest one day). On the 15nd day after inoculation (average tumor volume of mock group is over 1000 mm^3^), all the mice were sacrificed and dissected. Tumor volumes were measured for each group. The organs (heart, liver, spleen, lung, and kidney) were weighed and calculated the ratio of the organs to body. The blood samples were collected to further investigate the function of liver and kidney for the evaluation of subacute toxicity (UREA, Cr, ALT, AST).

**Tumor-targeted imaging**

Nude mice were divided into four groups randomly (three mice per group), including MLN-treated group with saline (100 μL qd), mock group with DOX, combination group of MLN (60 mg/Kg) and DOX, combination group of MLN and Gal-DOX. The combination groups preadministrated with MLN 10 days. Then, DOX or Gal-DOX was injected intraperitoneally into in a single dose of 5 mg/kg. Whole-body fluorescence images were continuously monitored after general anesthesia of mice at 0h, 1h, 6 h, 24 h, using an *in vivo* imaging system.

**Histology analysis**

Immunofluorescence was performed in frozen tissue sections. Whole tissue was fixed at RT for 15 min with a 4% formaldehyde solvent, permeabilized with 0.5% Triton™ X-100 for 3 min and subsequently blocked with Normal Donkey Serum for 1 hr at room temperature. They were then probed with anti-p21, Ki67 (ARG53222) antibodies at 4 ^o^C overnight. For TUNEL apoptosis analysis, the experiment was performed according to the manufacturer’s protocol (Beyotime, C1090). Nuclei were stained with DAPI (Thermo Fisher). The secondary antibodies were Alexa Fluor® 488 AffiniPure Donkey Anti-Rabbit IgG (H + L) (Jackson ImmunoResearch Laboratories, 71-545-152), Alexa Fluor® 488 AffiniPure Donkey Anti-Rat IgG (H + L) (Jackson ImmunoResearch Laboratories, 712-545-150), and Cy^TM3^ AffiniPure Donkey Anti-Rabbit IgG (H + L) (Jackson ImmunoResearch Laboratories, 711-165-152). Positive signal for p21, Ki67 and TUNEL was quantified with ImageJ.

**Statistical analysis**

Data are expressed as mean ± s.e.m. Group size was determined on the basis of the results of preliminary experiments and no statistical method was used to predetermine sample size. The indicated sample size (n = 3) represents biological replicates. Group allocation and outcome assessment were not performed in a blinded manner. All samples that met proper experimental conditions were included in the analysis. Statistical significance was determined by Student’s t-test using Prism 6 software (GraphPad Software). Significance was defined as **P* < 0.05, ***P* < 0.01, and ****P* < 0.001.


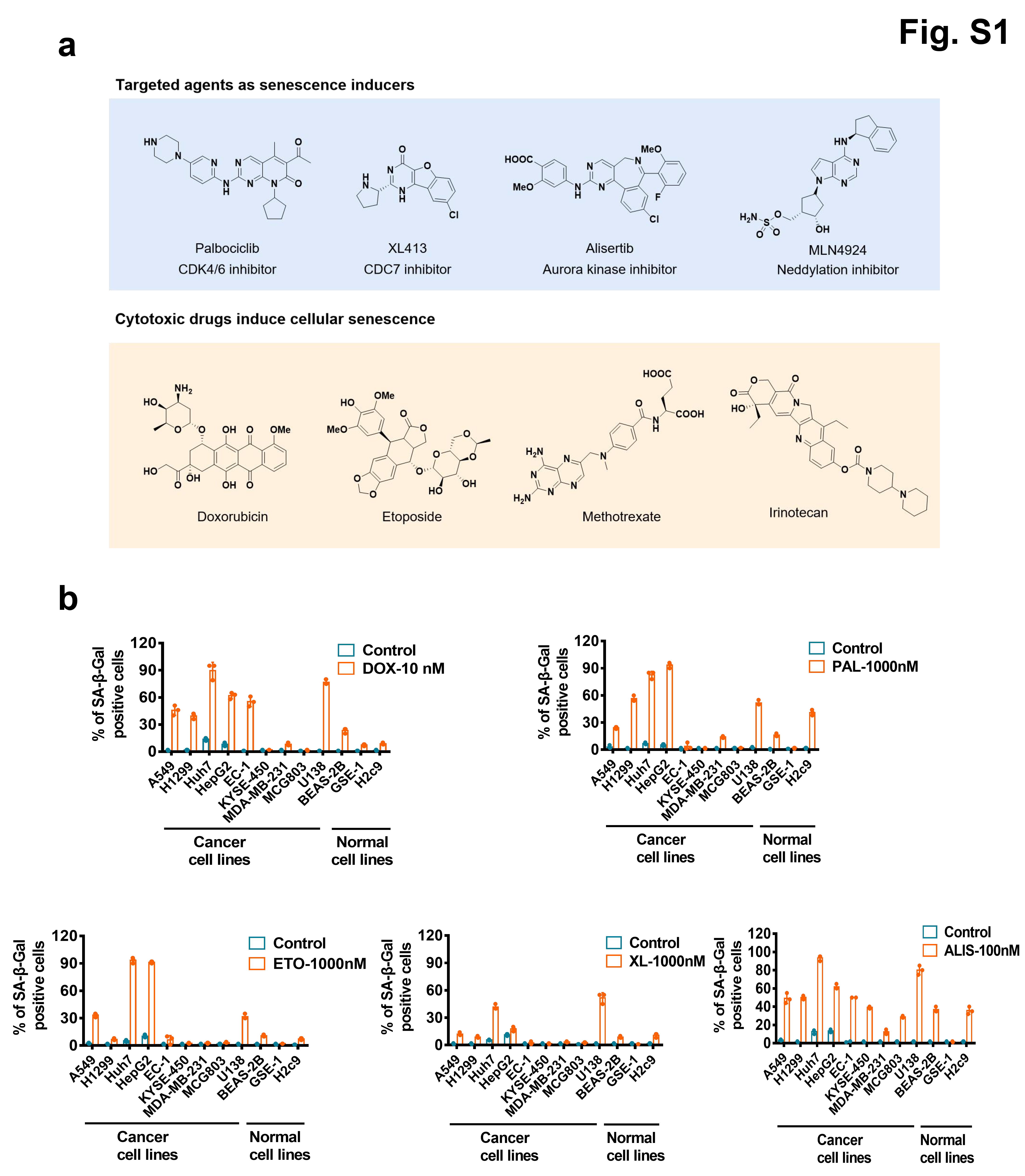


Figure. S1.

Evaluation of pro-senescence activity of MLN.

(**a**) Chemical structure of representative senescence inducers. (**b**) The SA-*β*-gal positive cells ratio of representative senescence inducers doxorubicin (DOX), palbociclib (PAL), etoposide (ETO), XL413 (XL) and alisertib (ALIS) in multiple cancer/normal cell lines.


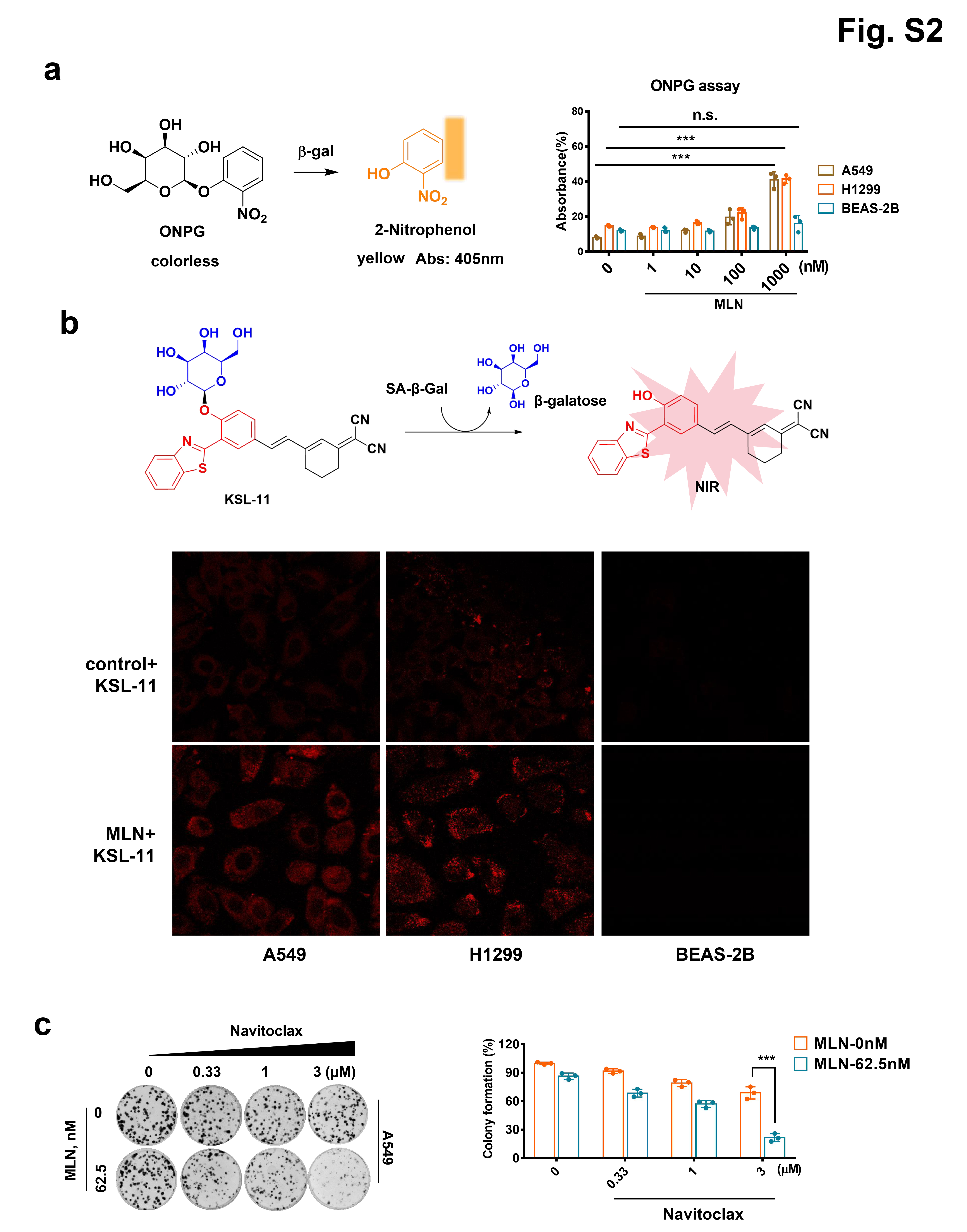


Figure. S2.

Assays evaluating the pro-senescence selectivity of MLN.

(**a**) Schematic diagram of ONPG color reaction assay and statistical data to explore the pro-senescence selectivity of MLN in A549, H1299 and BEAS-2B, respectively. (**b**) Fluorescence images of control or MLN treatment of A549, H1299, and BEAS-2B cell lines sequentially incubated with SA-*β*-gal responsive probe KSL11, respectively. (**c**) MLN-induced A549 senescent cancer cells were more sensitive to senolytic navitoclax than non-senescent A549 cells. Statistical significance was calculated with unpaired two-tailed Student’s t test. ****P* < 0.001, n.s. indicates no significant difference.


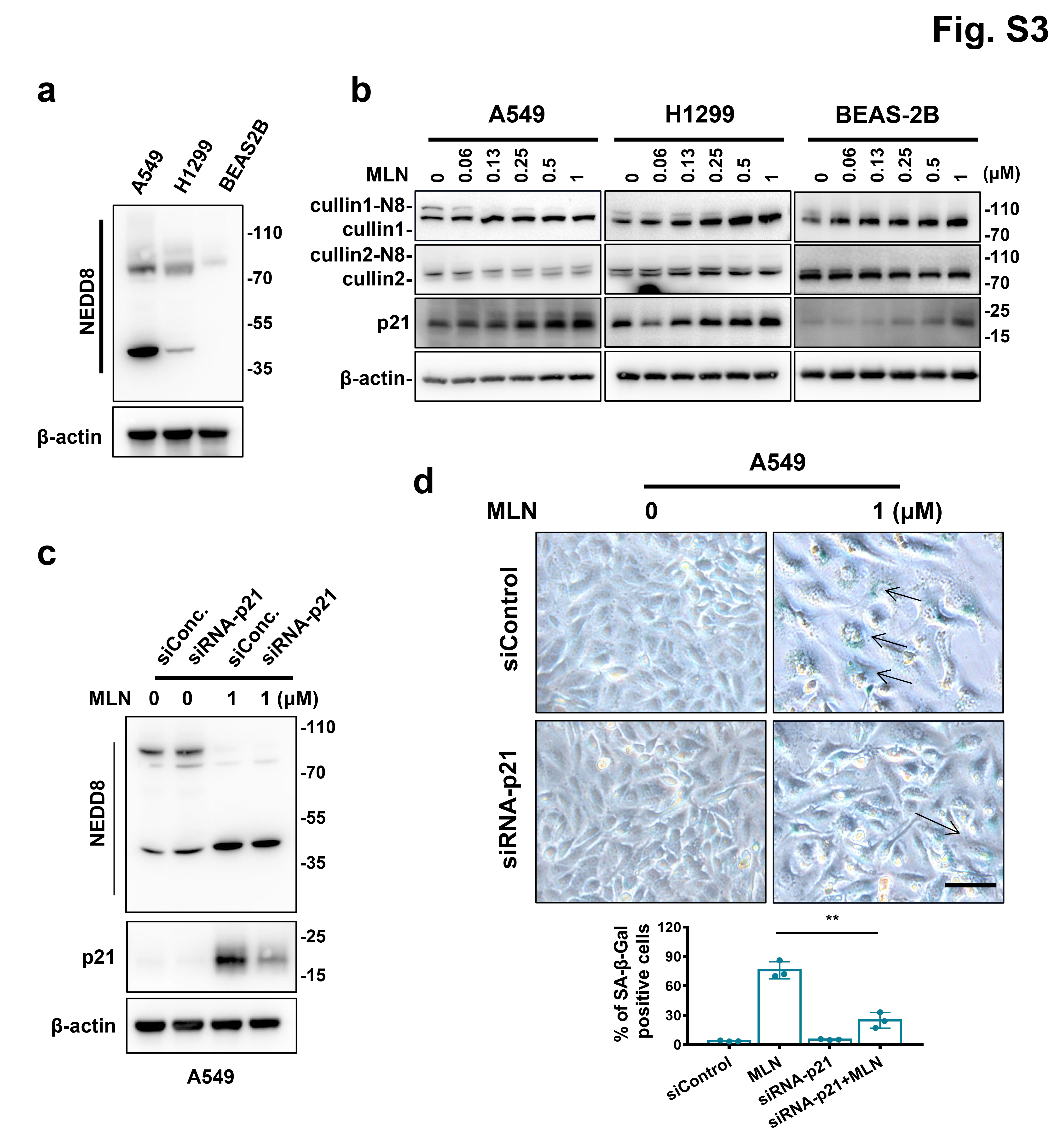


Figure. S3.

Pro-senescence selectivity of MLN positively related to the neddylation-dependent p21 accumulation.

(**a**) Investigation of NEDD8 modification of A549, H1299 and BEAS-2B by western blotting. (**b**) MLN treatment significantly induced the accumulation of classical neddylation substrate cullin1, cullin2 and the senescence marker p21 in A549 and H1299 cells, compared to BEAS-2B cells. (**c, d**) The downregulation of p21 decelerated the senescence in MLN-treated cancer cells. A549 cells transfected with siRNA-p21 were treated with MLN (1 μM) for 96 hours compared to control A549 cells and subjected to immunoblotting for NEDD8 with β-actin as a loading control (**c**) and SA-β-gal staining (**d**). Bar graphs represent percentage of SA-β-gal positive cell. Scale bar = 50 μm. Statistical significance was calculated with the unpaired two-tailed Student’s t tests. ***P* < 0.01.


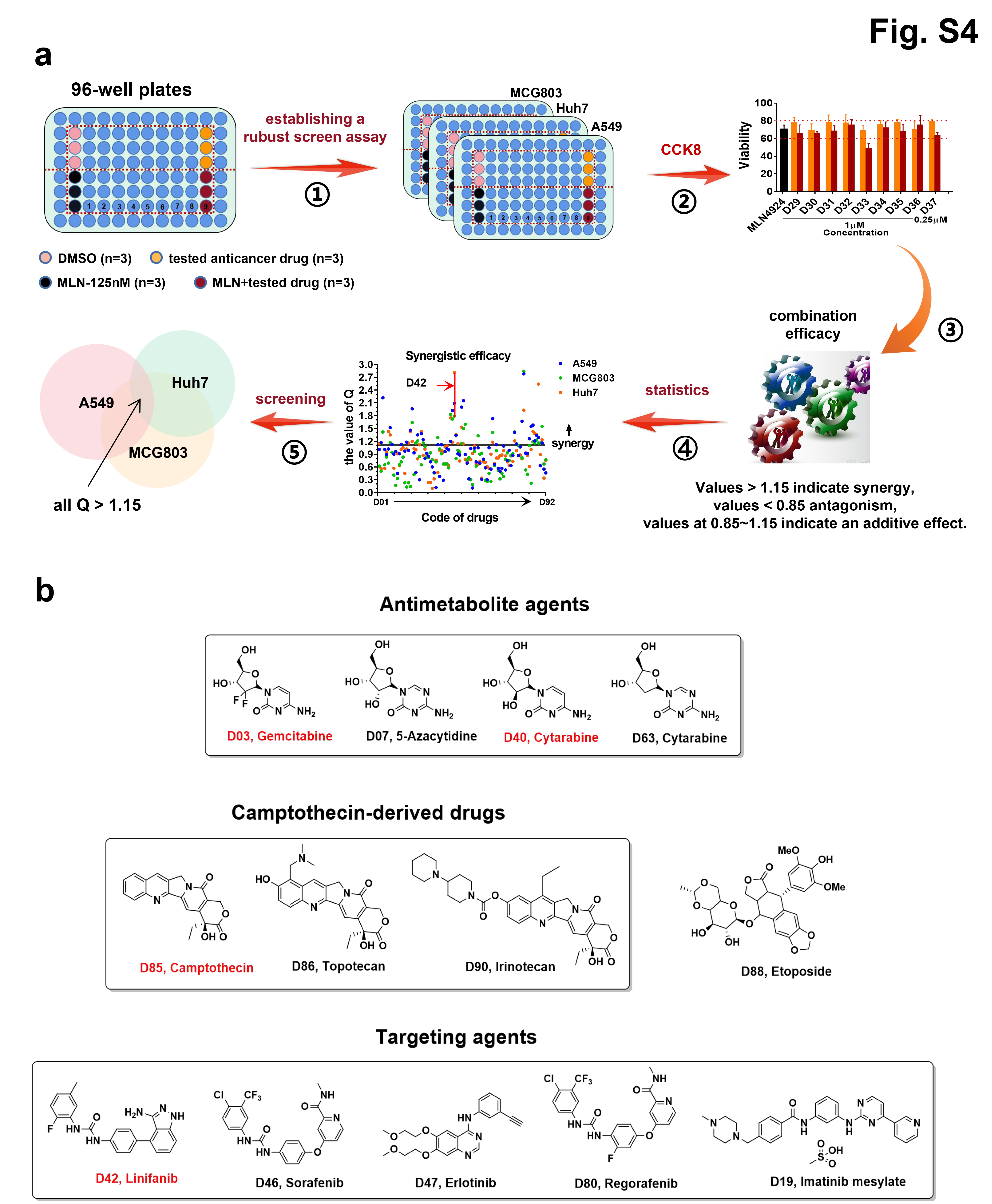


Figure. S4.

Screening and discovery of synergistic drugs with MLN.

(**a**) Flow diagram of synergistic drug screening (**b**) Structures of potential agents synergistic with MLN.


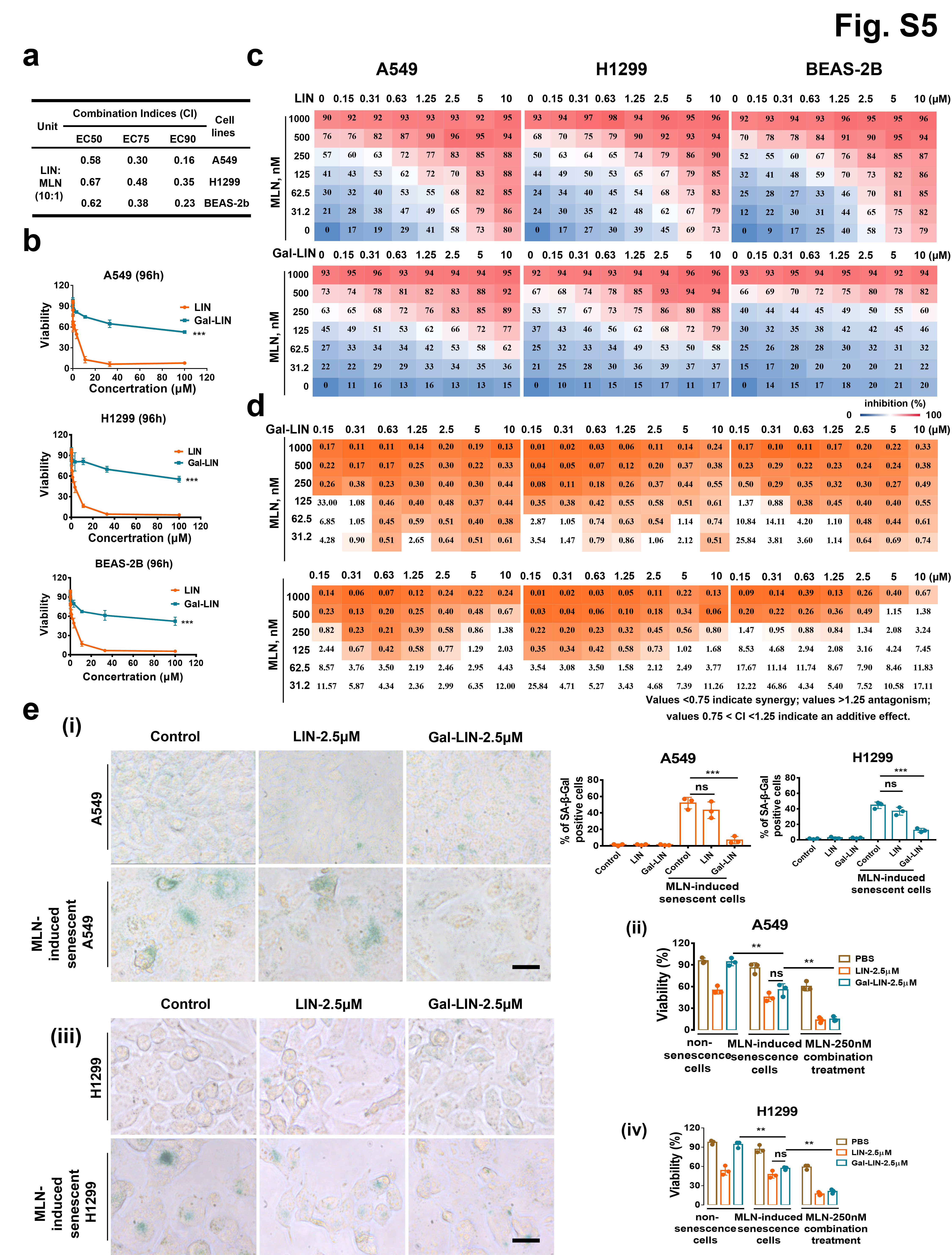




Figure. S5.

Gal-LIN as a novel bifunctional prodrug to selectively target cancer cells in combination with MLN.

(**a**) Combination indices of LIN (0.315-5 μM) and MLN4924 (31.5-500 nM) (the ratio of the two drugs was 10:1) were calculated by CalcuSyn. The concentration ratio of LIN and MLN was determined based on their respective GI_50_. (**b**) Survival curve of A549, H1299, and BEAS-2B cells treated with Gal-LIN, compared to LIN. (**c**) A549, H1299 and BEAS-2B cells were treated with escalating doses of MLN (31.2-1000 nM) with either LIN or Gal-LIN (0.15-10 μM) as indicated, followed by analysis of the cell proliferation rate; Percent inhibition of cell proliferation at each dose of drug is presented. (**d**) Qunatitative analysis of either LIN or Gal-LIN synergy in combination with MLN4924 on A549, H1299, and BEAS-2B cells. Drug synergism was analyzed using CompuSyn software (http://www.combosyn.com), which is based on the median-effect principle (Chou) and the combination index-isobologram theorem (Chou-Talay). CompuSyn software generates combination index (CI) values, where CI < 0.75 indicates synergism, CI = 0.75~1.25 indicates additive effects, and CI > 1.25 indicates antagonism. Following the software, drug combinations at non-constant ratios were used to calculate the combination index in our study. (**e**) Evaluation the elimination of senescence cells by Gal-LIN in MLN-induced senescent A549 and H1299 cells. Cells were pre-treated by MLN (250 nM), and then washed with PBS for determining the senolytic activity of Gal-LIN (2.5 μM) or LIN (2.5 μM) using SA-β-gal staining (i, iii). Investigation of the anti-proliferative efficacy of LIN or Gal-LIN in non-senescent / senescent cells, compared to the combinations of MLN with LIN or Gal-LIN alone with the CCK8 assay (ii, iv). Scale bar: 20 μm. (**f**) Evaluation of apoptosis of the combination treatment of MLN (250 nM) and Gal-LIN (2.5 μM), MLN (250 nM), Gal-LIN (2.5 μM) or control on A549 and H1299 cell lines evaluated with Annexin V/PI staining and flow cytometry. (**g**) Immunoblotting to determine the levels of cullin2, C-PARP, C-casp3 after the treatment with the combination of MLN and Gal-LIN, compared to MLN or LIN alone on A549 and H1299 cells, respectively. Statistical significance was calculated with the unpaired two-tailed Student’s t test. ***P* < 0.01, ****P* < 0.001, n.s. indicates no significant difference.


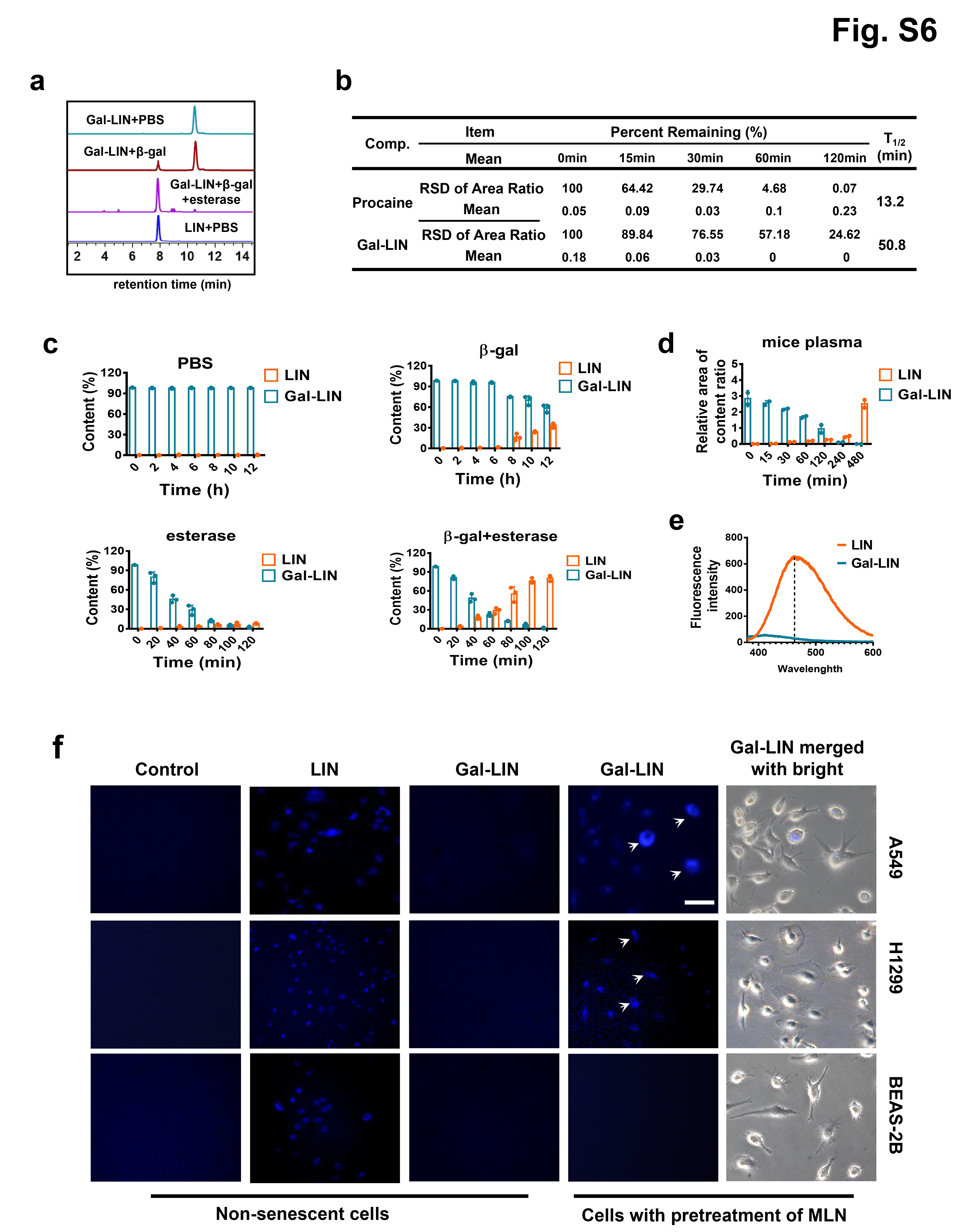


Figure. S6.

Evaluation of the stability and activation mode of Gal-LIN *in vitro* and in cells.

(**a**) HPLC profiles of Gal-LIN after incubation with PBS. A mixture of PBS with β-gal. A mixture of PBS with β-gal and esterase for 8h. Retention times: LIN (7.88 min), Gal-LIN (10.52 min). (**b, d**) Evaluation of stability of Gal-LIN in mice plasma and the relative area of content ratio by LC/MS/MS analysis. (**c**) Stability of Gal-LIN in various media and monitored by HPLC at t = 0, 2, 4, 6, 8, 10 and 12 h or 0, 20, 40, 60, 80, 100 and 120 min. (**e**) Fluorescence intentisy of LIN compared to Gal-LIN (Maximum absorption wavelength = 460 nm). (**f**) Fluorescence confocal microscopy showed that Gal-LIN (2.5 μM) is selectively activated with senescence A549 and H1299 cells. A549, H1299 and BEAS-2B cells were pre-treated with MLN (250 nM, 48 h), and then washed by PBS for testing the releasing selectivity of Gal-LIN. scale bar: 50 μm. Statistical significance was calculated with unpaired two-tailed Student’s t test. Data are mean values ± SEM (n = 3).


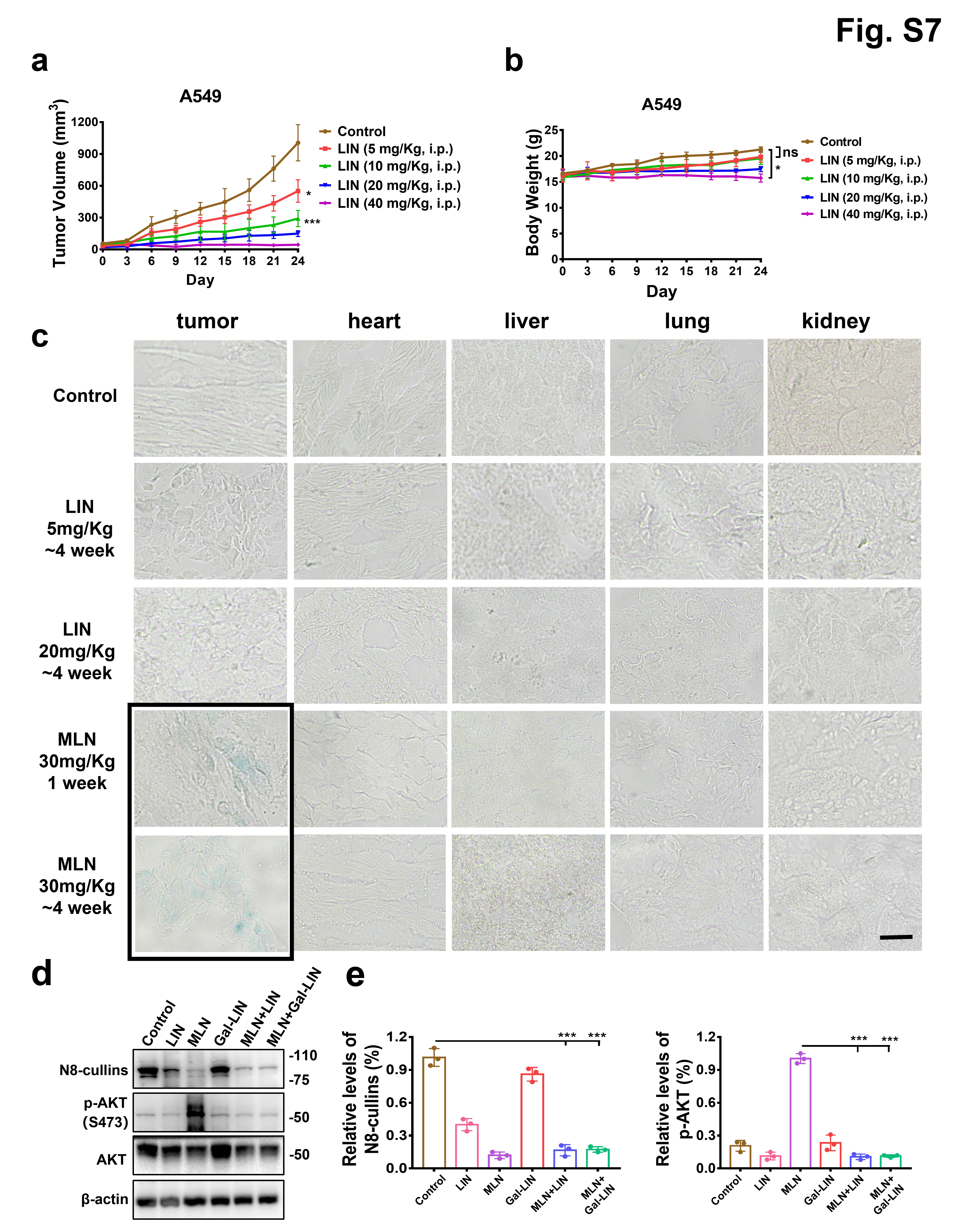


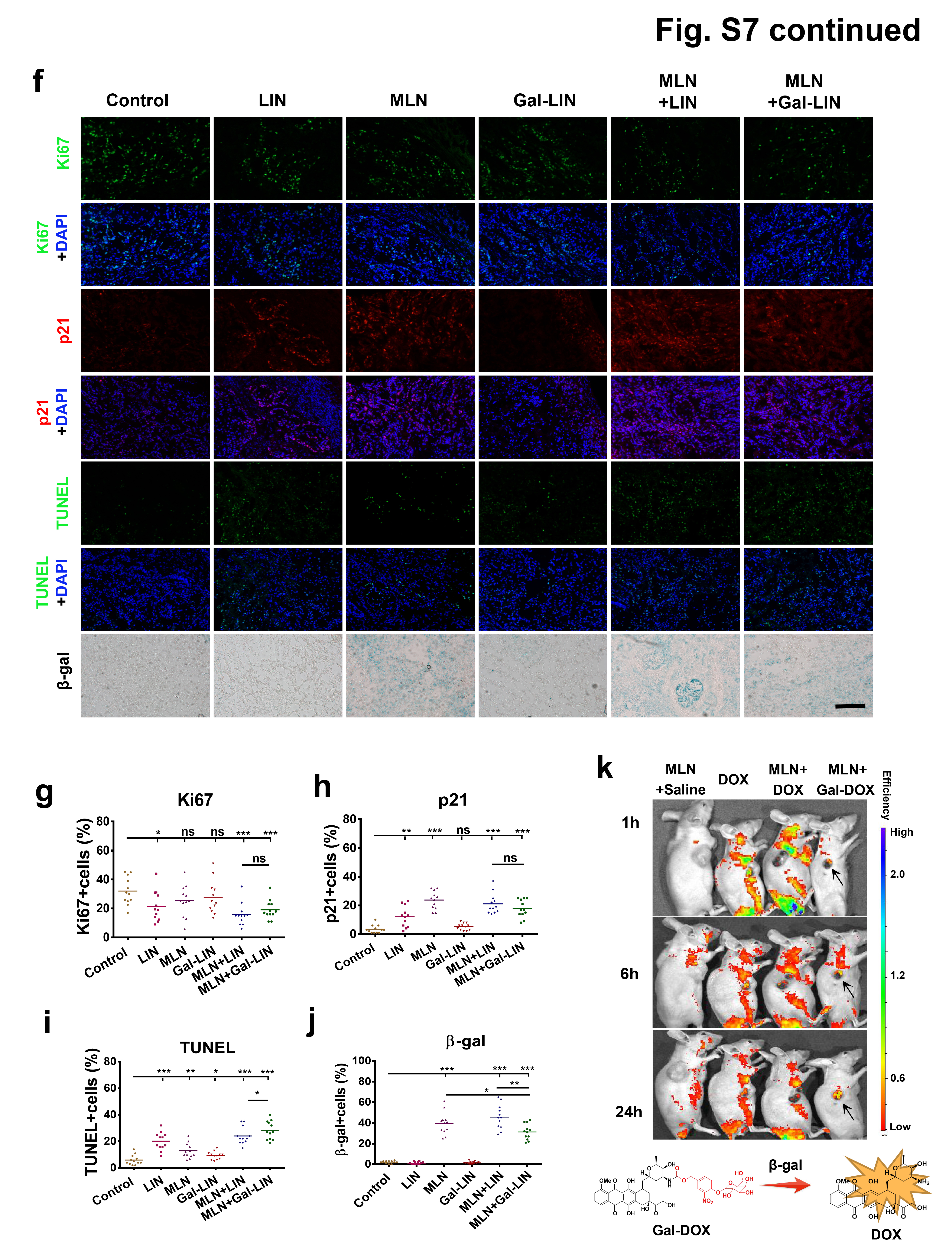


Figure. S7.

Combination treatment of MLN and Gal-LIN to suppress tumor growth with reduced toxicity.

Anticancer efficacy of LIN and pro-senescence time of MLN-treated A549 xenograft growth model: Tumor volume changed during treatment (**a**); body weight changed of mice during treatment (**b**); representative images of A549 xenografts and associated organs (heart, liver, lung and kidney) stained for SA-β-Gal activity (in blue) after treatment with various concentrations or time of LIN (5, 20 mg/Kg for 4 weeks), MLN (30 mg/Kg for 1 or 4 weeks) or vehicle. Scale bar = 200 μm (**c**). (**d, e**) Immunoblotting to determine the expression of p-AKT, AKT and cullins neddylation with β-actin as a loading control. (**f**) Representative histological images of tumors after the treatment of drugs alone or the combinations, stained for Ki67, p21 and β-gal expression, and labelled using TUNEL staining. Scale bar = 200 μm. Representative histological statistical analysis of tumors at the end of combination treatment, stained for Ki67 (**g**), p21 (**h**), TUNEL (**i**) and β-gal (**j**) expression. (**k**) In vivo fluorescence imaging of Gal-DOX-treated A549 bearing nude mice with the preadministration of MLN. Statistical significance was calculated with unpaired two-tailed Student’s t test. **P* < 0.05, ***P* < 0.01, ****P* < 0.001, n.s. indicates no significant difference.


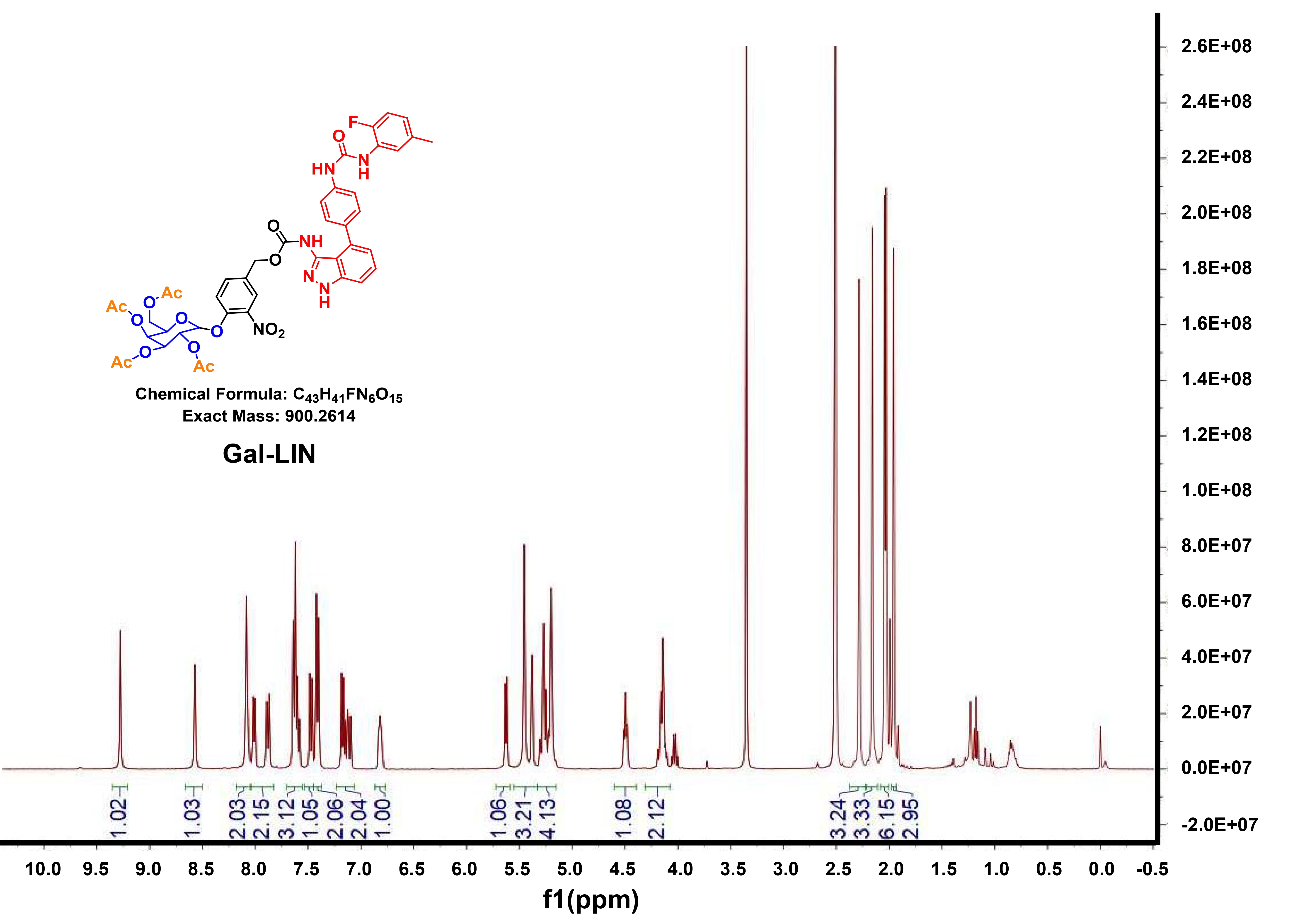


Figure. S8a.

HNMR of Gal-LIN.


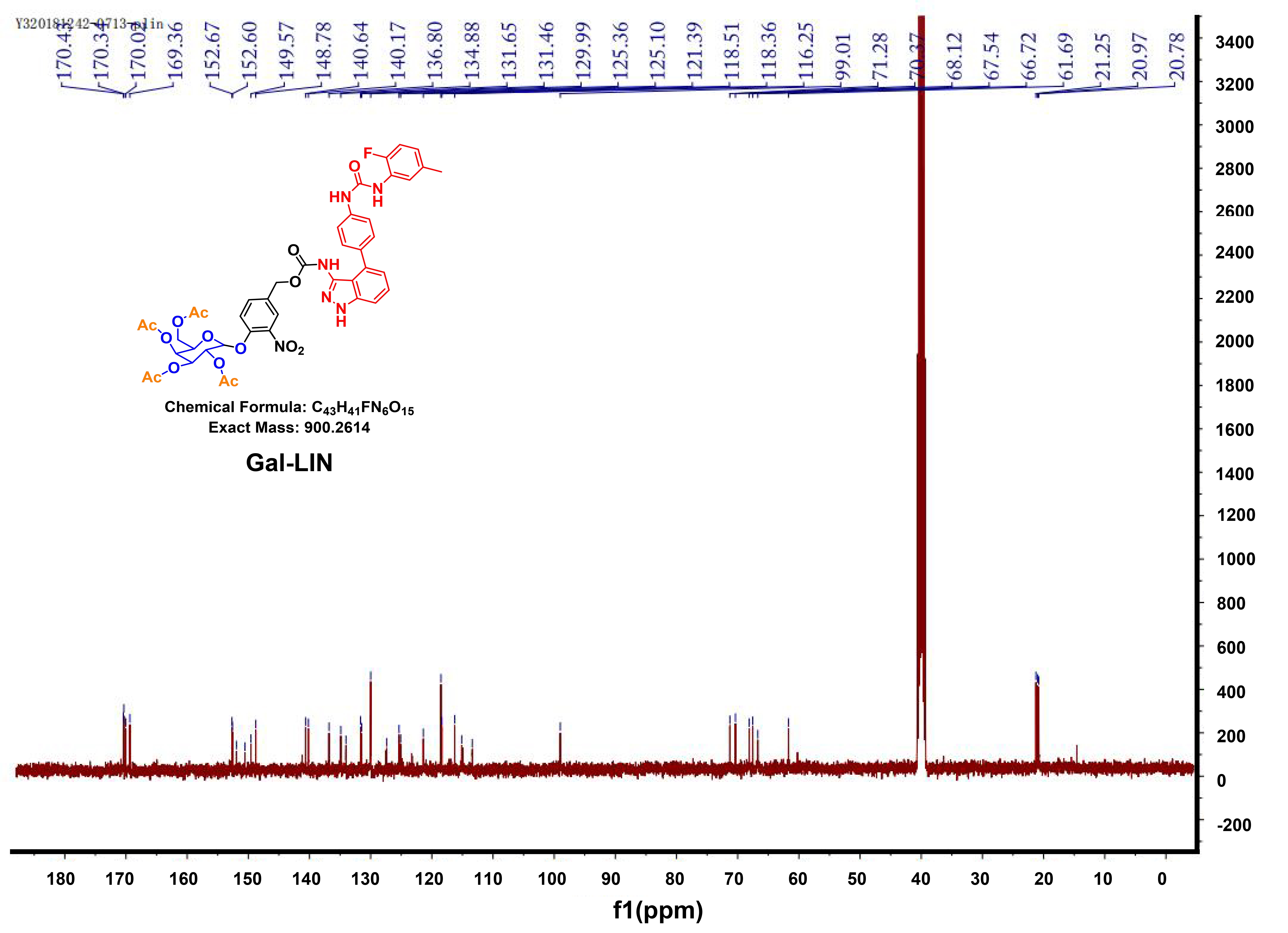


Figure. S8b.

CNMR of Gal-LIN.


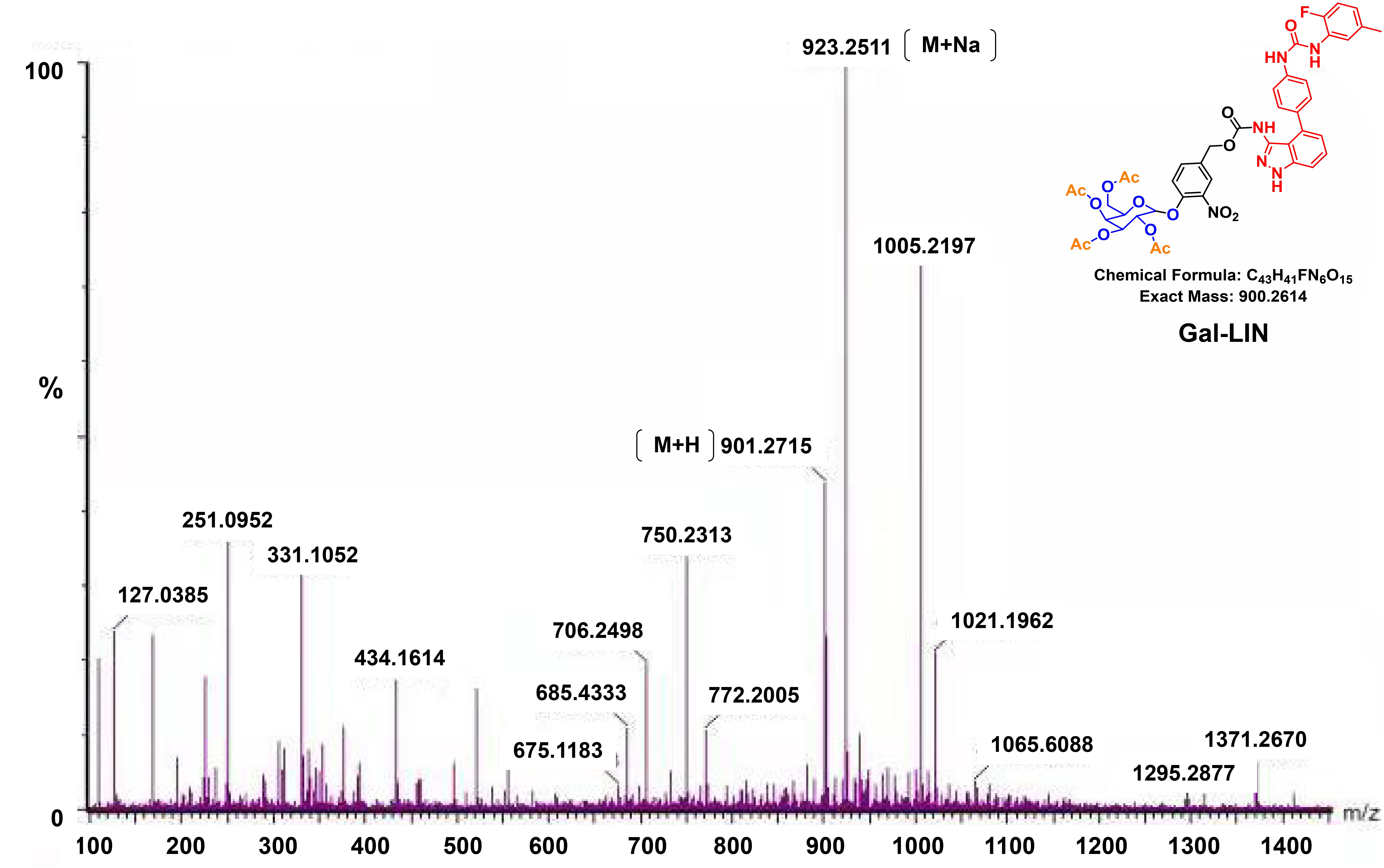


Figure. S8c.

ESI of Gal-LIN.

Table. S1.

Synergistic efficacy of neddylation inhibitor MLN with other anticancer agents in human lung cancer cell line A549, human gastric cancer cell MCG803, and human liver cancer cell line Huh7.

| **Number** | **Drug**  **Name** | **Anticancer**  **Type** | **Main**  **Target** | **Synergistic efficacy (Q value) *^a^*** | | |
| --- | --- | --- | --- | --- | --- | --- |
|  |  |  |  | **A549** | **MCG803** | **Huh7** |
| D01 | Methotrexate | Antimetabolite agents | DHFR | 1.01 | 0.62 | 1.15 |
| D02 | 5-FU | Antimetabolite agents | Pyrimidine | 1.17 | 0.50 | 0.56 |
| D03 | Gemcitabine | Antimetabolite agents | Pyrimidine | 2.22 | 1.42 | 3.10 |
| D04 | Busulfan | Alkylating agents | DNA | 1.01 | 0.65 | 0.57 |
| D05 | Thioguanine | Antimetabolite agents | Purine | 0.89 | 0.83 | 0.95 |
| D06 | Clofarabine | Antimetabolite agents | Purine | 1.02 | 0.69 | 1.43 |
| D07 | 5-AZA | Antimetabolite agents | Cytosine | 1.52 | 1.17 | 1.35 |
| D08 | Fludarabine phosphate | Antimetabolite agents | Purine | 1.13 | 0.94 | 0.94 |
| D09 | Hydroxyurea | Antimetabolite agents | Ribonucleotide Reductase | 0.90 | 0.41 | 0.57 |
| D10 | Miltefosine | Antimetabolite agents |  | 0.96 | 0.17 | 0.96 |
| D11 | Mercaptopurine | Antimetabolite agents | Purine | 0.45 | 0.33 | 0.91 |
| D12 | Temozolomide | Alkylating agents | DNA | 0.91 | 0.65 | 0.63 |
| D13 | Doxifluridine | Antimetabolite agents | Pyrimidine | 1.13 | 0.72 | 1.01 |
| D14 | Carmofur | Antimetabolite agents | Pyrimidine | 3.16 | 0.83 | 1.05 |
| D15 | Fludarabine | Antimetabolite agents | Purine | 0.97 | 0.23 | 0.45 |
| D16 | Dacarbazine | Alkylating agents | DNA | 1.18 | 0.13 | 1.02 |
| D17 | Abiraterone acetate | Hormone agents | CYP17 | 0.99 | 0.23 | 1.16 |
| D18 | Tamibarotene | Others | RARα | 1.46 | 0.14 | 1.43 |
| D19 | Imatinib mesylate | Targeting  agents | Tyrosine Kinase | 1.96 | 1.64 | 1.20 |
| D20 | Pemetrexed disodium | Antimetabolite agents | DHFR | 0.93 | 0.42 | 1.03 |
| D21 | Oxo (S-1) |  |  | 0.47 | 1.16 | 1.00 |
| D22 | FT (S-1) |  |  | 1.35 | 1.21 | 0.79 |
| D23 | CDHP (S-1) |  |  | 0.30 | 0.84 | 1.12 |
| D24 | Tamoxifen | Hormone agents | Antiestrogen | 1.38 | 0.65 | 1.43 |
| D25 | Tamoxifen citrate | Hormone agents | Antiestrogen | 1.24 | 1.16 | 0.87 |
| D26 | Letrozole | Hormone agents | Aromatase | 0.80 | 1.08 | 1.02 |
| D27 | Vorinostat | Targeting  agents | HDAC | 0.92 | 0.84 | 0.89 |
| D28 | Zoledronic acid | Others | Mevalonate Pathway | 0.85 | 0.82 | 0.24 |
| D29 | Cisplatin | Metal agents | DNA | 0.72 | 0.93 | 0.24 |
| D30 | Toremifene citrate | Hormone agents | Antiestrogen | 0.60 | 0.77 | 0.10 |
| D31 | Altretamine | Antimetabolite agents | DHFR | 0.47 | 0.46 | 0.32 |
| D32 | Isophosphamide | Alkylating agents | DNA | 0.12 | 0.72 | 0.76 |
| D33 | Oxaliplatin | Metal agents | DNA | 1.12 | 1.29 | 0.47 |
| D34 | Capecitabine | Antimetabolite agents | Pyrimidine | 0.38 | 1.02 | 0.99 |
| D35 | Aminoglutethimide | Hormone agents | cholesterol | 1.12 | 0.91 | 0.99 |
| D36 | Carboplatin | Metal agents | DNA | 0.29 | 0.67 | 0.95 |
| D37 | Azathioprine | Antimetabolite agents | Purine | 0.96 | 0.67 | 1.07 |
| D38 | Thiotepa | Alkylating agents | DNA | 1.03 | 0.67 | 1.09 |
| D39 | Cyclophosphamide | Alkylating agents | DNA | 1.00 | 0.58 | 0.63 |
| D40 | Cytarabine | Antimetabolite agents | Cytosine | 1.20 | 1.83 | 1.75 |
| D41 | Cytarabine hydrochloride | Antimetabolite agents | Cytosine | 1.92 | 1.72 | 1.18 |
| D42 | Linifanib | Targeting  agents | Tyrosine Kinase | 2.09 | 1.79 | 2.81 |
| D43 | Pazopanib | Targeting  agents | Tyrosine Kinase | 1.34 | 0.80 | 1.36 |
| D44 | Crizotinib | Targeting  agents | ALK、c-MET、ROS1 | 0.91 | 0.99 | 1.15 |
| D45 | Gefitinib | Targeting  agents | Tyrosine Kinase | 0.88 | 0.95 | 0.74 |
| D46 | Sorafenib tosylate | Targeting  agents | Tyrosine Kinase | 2.00 | 1.15 | 1.53 |
| D47 | Erlotinib hydrochloride | Targeting  agents | Tyrosine Kinase | 2.15 | 1.50 | 1.14 |
| D48 | Dasatinib monohydrate | Targeting  agents | Tyrosine Kinase | 0.89 | 1.60 | 0.78 |
| D49 | 6-Mercaptopurine monohydrate | Antimetabolite agents | Purine | 0.69 | 0.76 | 0.72 |
| D50 | Anastrozole | Hormone agents | Aromatase | 0.32 | 0.82 | 0.80 |
| D51 | Bosutinib | Targeting  agents | Tyrosine Kinase | 1.03 | 0.30 | 0.77 |
| D52 | Tipiracil | Antimetabolite agents | Pyrimidine | 0.11 | 0.19 | 0.92 |
| D53 | Nintedanib Ethanesulfonate Salt | Targeting  agents | Tyrosine Kinase | 1.04 | 0.96 | 1.09 |
| D54 | Olaparib | Targeting  agents | PARP | 0.72 | 0.62 | 0.67 |
| D55 | Ancitabine hydrochloride | Antimetabolite agents | Cytosine | 1.05 | 1.33 | 0.66 |
| D56 | FUDR | Antimetabolite agents | Pyrimidine | 0.69 | 0.90 | 0.73 |
| D57 | Flutamide | Hormone agents | Androgen | 0.31 | 0.92 | 0.34 |
| D58 | Chloroambucil | Alkylating agents | DNA | 0.78 | 0.77 | 0.72 |
| D59 | Ctimonacic |  | Other | 0.58 | 0.29 | 0.71 |
| D60 | Medroxyprogesterone Acetate | Hormone agents | Progestogens | 0.30 | 0.26 | 0.61 |
| D61 | Catharanthine sulfate | Natural product agents |  | 1.05 | 0.45 | 0.57 |
| D62 | Procarbazine hydrochloride | Others | DNA | 0.55 | 0.26 | 0.90 |
| D63 | 5-Aza-2'-deoxycytidine | Antimetabolite agents | Pyrimidine | 1.73 | 1.41 | 1.18 |
| D64 | Vandetanib | Targeting  agents | Tyrosine Kinase | 0.66 | 0.63 | 0.68 |
| D65 | Carmustine | Alkylating agents | DNA | 1.12 | 0.98 | 0.81 |
| D66 | Melphalan | Alkylating agents | DNA | 0.57 | 0.52 | 0.81 |
| D67 | Pirfenidone | Others |  | 0.50 | 0.32 | 0.86 |
| D68 | Lenalidomide | Targeting  agents | Proteasome | 0.57 | 0.45 | 0.88 |
| D69 | Exemestane | Hormone agents | Aromatase | 1.09 | 0.38 | 0.98 |
| D70 | Lomustine | Alkylating agents | DNA | 0.53 | 0.78 | 0.86 |
| D71 | Sunitinib Malate | Targeting  agents | Tyrosine Kinase | 0.75 | 0.93 | 0.72 |
| D72 | Mitoxantrone hydrochloride | Antibody agents |  | 0.99 | 0.75 | 0.64 |
| D73 | Dexrazoxane hydrochloride | Other |  | 0.63 | 0.87 | 0.86 |
| D74 | Palbociclib | Targeting  agents | CDK4/6 | 0.87 | 0.91 | 0.73 |
| D75 | Lapatinib ditosylate | Targeting  agents | Tyrosine Kinase | 1.41 | 1.42 | 1.21 |
| D76 | Formestane | Hormone agents | Aromatase | 1.14 | 1.14 | 0.98 |
| D77 | bepotastine benzenesulfonate | Alkylating agents | DNA | 0.87 | 1.10 | 0.87 |
| D78 | Entinostat | Targeting  agents | HDAC | 1.07 | 1.07 | 0.88 |
| D79 | lenvatinib Mesylate | Targeting  agents | Tyrosine Kinase | 1.67 | 0.81 | 0.79 |
| D80 | Regorafenib | Targeting  agents | Tyrosine Kinase | 2.78 | 2.84 | 1.93 |
| D81 | Epirubicin hydrochloride | Antibody agents |  | 0.92 | 0.53 | 0.93 |
| D82 | Doxorubicin hydrochloride | Antibody agents |  | 0.911 | 0.43 | 0.87 |
| D83 | Ibrutinib | Targeting  agents | BTK | 1.26 | 1.10 | 0.61 |
| D84 | protocatechnic aldehyde | Natural product agents |  | 1.55 | 0.22 | 1.01 |
| D85 | Camptothecin | Natural product agents | TOPOI | 1.58 | 1.19 | 1.43 |
| D86 | Topotecan | Natural product agents | TOPOI | 1.21 | 1.16 | 1.38 |
| D87 | Topotecan  hydrochloride | Natural product agents | TOPOI | 1.34 | 1.23 | 1.36 |
| D88 | Etoposide | Natural product agents | TOPOII | 1.29 | 1.16 | 2.54 |
| D89 | Betulin | Natural product agents |  | 0.63 | 1.16 | 0.89 |
| D90 | Irinotecan hydrochloride | Natural product agents |  | 1.16 | 1.55 | 1.24 |
| D91 | Paclitaxel | Natural product agents |  | 0.55 | 0.67 | 0.34 |
| D92 | Taxotere | Natural product agents |  | 0.46 | 0.77 | 0.75 |

*^a^*Jin's formula was used to analyse the effect of drug combination.

$$\text{Q}=\text{E}_{\text{A}+\text{B}}\text{/（E}_{\text{A}}+\text{E}_{\text{B}}\text{-E}_{\text{A}}\times\text{E}_{\text{B}}\text{）}$$

Q represents the value of combination efficacy. Herein, E_A+B_ represents cellular inhibitory ratio of combination; E_A_ represents cellular inhibitory ratio of drug A; E_B_ represents cellular inhibitory ratio of drug B; Values > 1.15 indicate synergy, values < 0.85 antagonism, values at 0.85~1.15 indicate an additive effect.


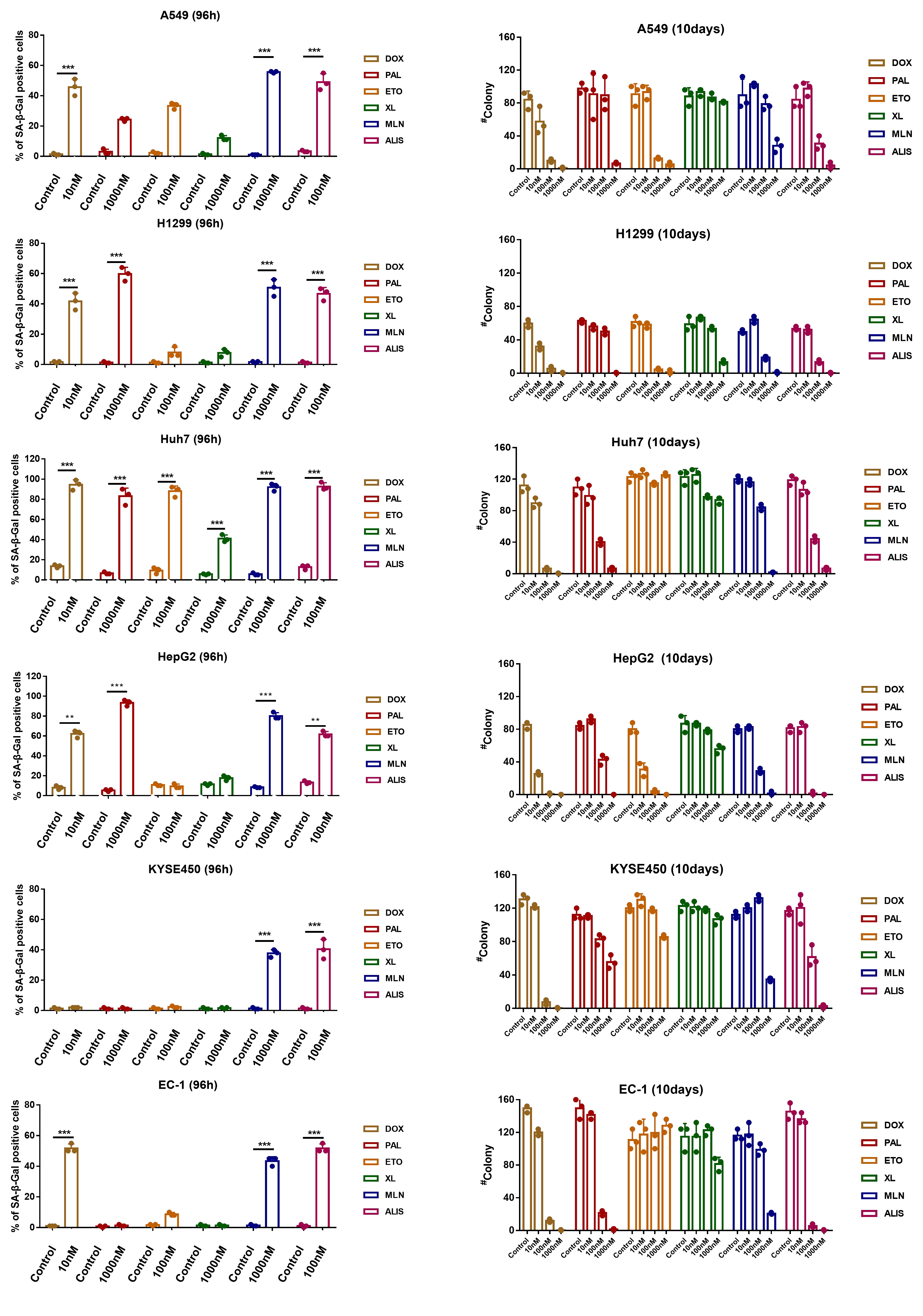

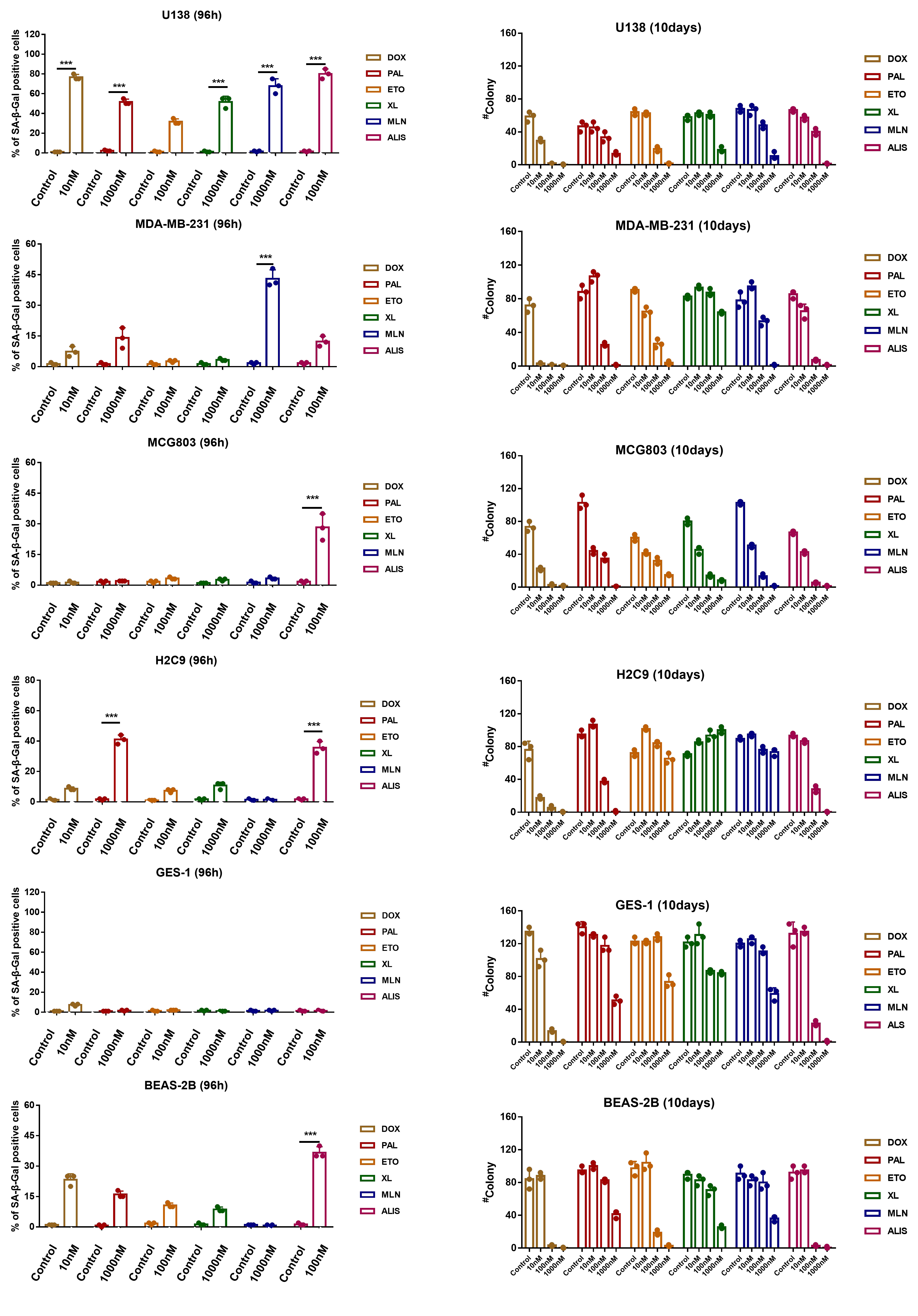


Supplementary Materials of Figure S1.

SA-*β*-galactosidase activities and [colony formation](http://www.baidu.com/link?url=hmLvibW5v5P_-NBmXQwlmYObvx79j2ww0nAsDjUI1ARhrkuXUvc2VJpahS4wM_nqxkG6rdWjaDVDA9jTvwrjqcgIWjRgRbv8sBtrn16b72uCez_kVkS40f6XgaoZI_pG)s of senescence inducers (DOX, PAL, ETO, XL, MLN, ALIS) in human lung cancer cell A549 and H1299, human liver cancer cell HepG2 and Huh7, human esophageal cancer cell KYSE450 and EC-1, human glioma cell U138, human gastric cancer cell MCG803, human breast cancer cell MDA-MB-231, rat cardiacmyoblast H2c9, human lung cell BEAS-2B, human gastric cell GES-1, respectively. SA-*β*-galactosidase activity of treated A549 cells at 96h. Senescent cells are characterized by accumulation of SA-*β*-gal, as well as enlarged size and flattened morphology (left). The panels of various cancer cells were seeded at low confluence and grown in the absence or presence of representative senescence inducers (DOX, PAL, ETO, XL, MLN, ALIS) at various concentrations (10, 100, 1000 nM) for 10 d (right). Statistical significance was calculated with unpaired two-tailed Student’s t test. ***P* < 0.01, ****P* < 0.001.
